# Supplementary material for: An empirical evaluation of four variants of a universal species–area relationship
Source: PeerJ. 2013 Nov 21;1:e212. doi: 10.7717/peerj.212 (PMC3840416; doi:10.7717/peerj.212)
Supplement: Table S1 — A more complete description of the datasets used to test the spatial predictions of METE. [file peerj-01-212-s001.doc]

| **Site names** | **lat** | **long** | **habitat** | **ref** | **years since dist** | **year of census** | **DBH cutoff (mm)** | **shape** | ***A*0­ (ha)** | ***S*0** | ***N*0** |
| --- | --- | --- | --- | --- | --- | --- | --- | --- | --- | --- | --- |
| BCI | 9.154 | -79.846 | mature lowland moist tropical forest | 1-3 | 500 | 2010 | 10 | rectangle | 50 | 301 | 205096 |
| Sherman1 | 9.364 | -79.955 | mature mixed secondary lowland moist tropical forest | 4 | 200 | 1999 | 10 | rectangle | 2 | 181 | 7502 |
| Sherman2 |  |  |  |  |  |  |  |  |  | 168 | 7743 |
| Cocoli1 | 8.976 | -79.592 | secondary-to-mature lowland dry tropical forest | 4 | 100 | 1998 | 10 | rectangle | 2 | 136 | 4411 |
| Cocoli2 |  |  |  |  |  |  |  |  |  | 141 | 4241 |
| Luquillo | 18.326 | -65.816 | disturbed tropical forest | 5 | 12 | 2006 | 10 | rectangle | 12.5 | 124 | 32320 |
| Bryan | 36.000 | -78.943 | almost all-aged hardwoods; acid soils | 6-8 | 500 | 1991 | 10 | rectangle | 1.71125 | 48 | 3394 |
| Big Oak | 35.882 | -79.013 | all-aged bottomland hardwoods | 6-8 | 500 | 1993 | 10 | rectangle | 2 | 40 | 5469 |
| Oosting | 35.976 | -79.057 | all-aged hardwoods; circumneural soils | 9 | 500 | 1990 | 20 | square | 6.5536 | 39 | 8892 |
| Rocky | 35.986 | -79.036 | all-aged hardwoods; circumneural soils | 6-8 | 500 | 1990 | 10 | square | 1.44 | 37 | 3383 |
| Bormann | 36.021 | -78.998 | all-aged hardwoods; acid soils | 6-8 | 500 | 1993 | 10 | square | 1.96 | 30 | 3879 |
| Wood Bridge | 35.981 | -79.030 | almost all-aged hardwoods; acid soils | 6-8 | 500 | 1991 | 10 | square | 0.5041 | 19 | 758 |
| Bald Mnt | 35.976 | -79.111 | all-aged hardwoods; extremely acid soils | 6-8 | 500 | 1991 | 10 | rectangle | 0.5 | 17 | 669 |
| Landsend | 35.989 | -79.032 | Old field pine | 6-8 | 95 | 1993 | 10 | rectangle | 0.845 | 41 | 2139 |
| Graveyard | 35.993 | -79.036 | Old field pine | 6-8 | 75 | 1992 | 10 | square | 1 | 36 | 2584 |
| UCSC | 37.012 | -122.075 | Mediterranean, mixed evergreen coastal forest | 10 | 70 | 2007 | 10 | rectangle | 4.5 | 31 | 5885 |
| Serpentine | 38.865 | -122.413 | annual serpentine grassland | 11 |  | 1998 | NA | square | 0.0064 | 24 | 37182 |
| Crosstimbers | 36.838 | -96.414 | oak woodland | 12 | 1 | 1998 | 25 | square | 4 | 7 | 7625 |

1. Condit (1998), 2. Hubbell et al. (1999), 3. Hubbell et al. (2005), 4. Condit et al. (2004), 5. Zimmerman et al. (1994), 6. Peet and Christensen (1987), 7. McDonald et al. (2002), 8. Xi et al. (2008), 9. Palmer et al. (2007), 10. Gilbert et al. (2010), 11. Green et al. (2003), 12. Arévalo (2013)

**References**

1. Condit, R. 1998. Tropical Forest Census Plots. Springer-Verlag and R. G. Landes Company, Berlin, Germany and Georgetown, Texas.
2. Hubbell, S. P., R. B. Foster, S. T. O’Brien, K. E. Harms, R. Condit, B. Wechsler, S. J. Wright, and S. L. de Lao. 1999. Light-gap disturbances, recruitment limitation, and tree diversity in a neotropical forest. Science 283:554–557.
3. Hubbell, S. P., R. Condit, and R. B. Foster. 2005. Barro Colorado Forest Census Plot Data. URL http://ctfs.arnarb.harvard.edu/webatlas/datasets/bci.
4. Condit, R., Salomon Aguilar, A. Hernandez, R. Perez, S. Lao, G. Angehr, S. P. Hubbell, and R. B. Foster. 2004. Tropical Forest Dynamics across a Rainfall Gradient and the Impact of an El Niño Dry Season. Journal of Tropical Ecology 20:51–72.
5. Zimmerman, J. K., E. M. Everham, III, R. B. Waide, D. J. Lodge, C. M. Taylor, and N. V. L. Brokaw. 1994. Responses of tree species to hurricane winds in subtropical wet forest in Puerto Rico: implications for tropical tree life histories. Journal of Ecology 82:911–922.
6. Peet, R. K., and N. L. Christensen. 1987. Competition and Tree Death. BioScience 37:586–595.
7. McDonald, R. I., R. K. Peet, and D. L. Urban. 2002. Environmental correlates of oak decline and red maple increase in the North Carolina piedmont. Castanea 67:84–95.
8. Xi, W., R. K. Peet, J. K. Decoster, and D. L. Urban. 2008. Tree damage risk factors associated with large, infrequent wind disturbances of Carolina forests. Forestry 81:317–334.
9. Palmer, M. W., R. K. Peet, R. A. Reed, W. Xi, and P. S. White. 2007. A multiscale study of vascular plants in a North Carolina piedmont forest. Ecology 88:2674.
10. Gilbert, G. S., E. Howard, B. Ayala-Orozco, M. Bonilla-Moheno, J. Cummings, S. Langridge, I. M. Parker, J. Pasari, D. Schweizer, and S. Swope. 2010. Beyond the tropics: forest structure in a temperate forest mapped plot. Journal of Vegetation Science 21:388–405.
11. Green, J. L., J. Harte, and A. Ostling. 2003. Species richness, endemism and abundance patterns: tests of two fractal models in a serpentine grassland. Ecology Letters 6:919–928.
12. Arévalo, J. R. 2013. Spatial analysis and structure of a cross-timber stand in the TallGrass Prairie Preserve (Pawhuska, Oklahoma). Journal of Forestry Research 24:47–52.
