# Supplementary figures and images for: An empirical evaluation of four variants of a universal species–area relationship
Source: PeerJ. 2013 Nov 21;1:e212. doi: 10.7717/peerj.212 (PMC3840416; doi:10.7717/peerj.212)

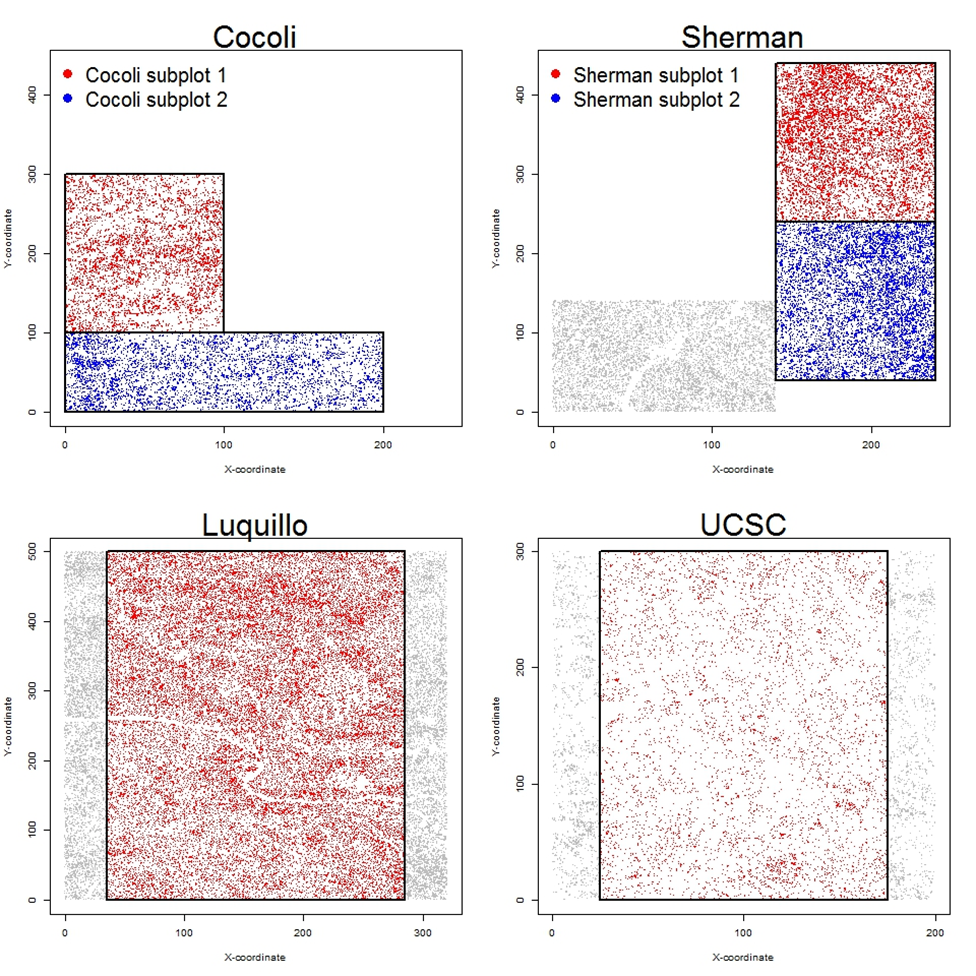

Supplement: Figure S1 — Stem maps for four of the study sites that required subsampling. The maps illustrate how the spatial data was partitioned for the analysis. Each dot represents a stem record, the colored stems were included in the analysis and the grey stems were not. Note that the dimensions of the plots appear visually distorted, but in reality they are all rectangles with an aspect ratio of 2:1. [file peerj-01-212-s002.png]

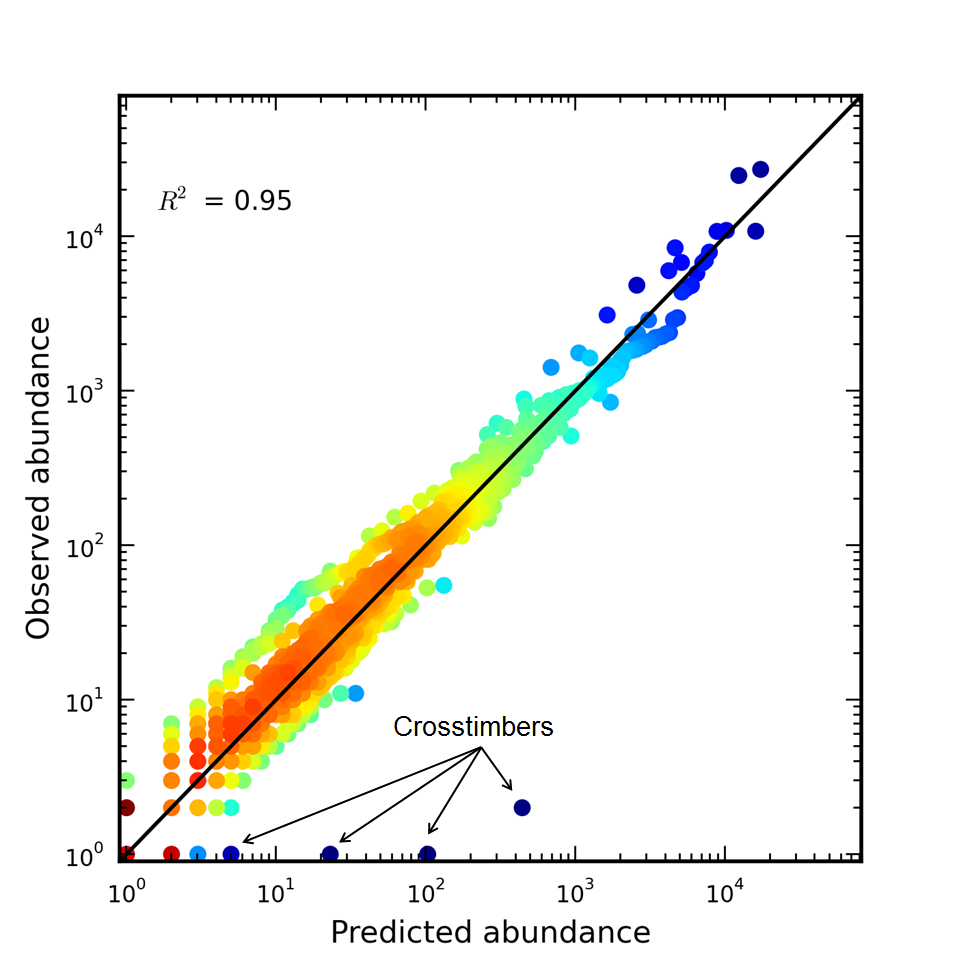

Supplement: Figure S2 — The observed-predicted plot for the species abundance distribution (SAD) across all 16 communities. The line is the 1:1 line. The points are color-coded to reflect the density of neighboring points, with warm (red) colors representing higher densities and cold (blue) colors representing lower densities. [file peerj-01-212-s003.png]
